# Individualized Cerebral Artery Protection Strategies for the Surgical Treatment of Parasellar Meningiomas on the Basis of Preoperative Imaging

**DOI:** 10.3389/fonc.2021.771431

**Published:** 2021-12-02

**Authors:** Yang Li, XingShu Zhang, Jun Su, Chaoying Qin, Xiangyu Wang, Kai Xiao, Qing Liu

**Affiliations:** ^1^ Department of Neurosurgery in Xiangya Hospital, Central South University, Changsha, China; ^2^ Department of Neurosurgery in Hunan Children’s Hospital, Changsha, China

**Keywords:** parasellar meningioma, imaging, perforating artery, skull base surgery, KPS

## Abstract

**Objective:**

Parasellar meningiomas (PMs) represent a cohort of skull base tumors that are localized in the parasellar region. PMs tend to compress, encase, or even invade the cerebral arteries and their perforating branches. The surgical resection of PMs without damaging neurovascular structures is challenging. This study aimed to analyze functional outcomes in a series of patients who underwent surgery with individualized cerebral artery protection strategies based on preoperative imaging.

**Methods:**

A retrospective review was performed on a single surgeon’s experience of the microsurgical removal of PMs in 163 patients between January 2012 and March 2020. Individualized approaches with a bidirectional dissection strategy were used. Cerebral artery invasion classification, neurological outcomes, MRC Scale for muscle strength, and Karnofsky performance scale were used to assess tumor vascular invasion, functional outcome, and patient quality-of-life outcomes, respectively.

**Results:**

Total resection (Simpson grade I or II) was achieved in 114 patients (69.9%) in our study. A total of 44.7% of patients had improved vision at consecutive follow-ups, 51.1% were stable, and 3.8% deteriorated. Improvements in cranial nerves III, IV, and VI were observed in 41.1%, 36.2%, and 44.8% of patients, respectively. The mean follow-up time was (38.8 ± 27.9) months, and the KPS at the last follow-up was 89.6 ± 8.5. Recurrence was observed in eight patients (13.8%) with cavernous sinus meningiomas, and the recurrence rates in anterior clinoid meningiomas and medial sphenoid wing meningiomas were 3.8% and 2.8%, respectively.

**Conclusions:**

Preoperative imaging is important in the selection of surgical approaches. Maximum tumor resection and cerebral artery protection can be achieved concurrently by utilizing the bidirectional dissection technique. Individualized cerebral artery protection strategies provide great utility in improving a patient’s quality of life.

## Introduction

Parasellar meningiomas (PMs) are a heterogeneous group of tumors that originate in the parasellar region. They frequently compress, encase, or even invade adjacent neurovascular structures of the anterior and middle skull base, thus making their surgical management challenging for skull base surgeons. However, a clear definition of PMs as a distinct entity is lacking because their multiple definitions and classification. Stirling (1) first described PMs in 1896. Ugrumov (2) divided PMs into three subgroups according to their site of origin: anterior clinoid meningiomas(ACMs), medial sphenoid wing meningiomas (MSWMs), and cavernous sinus meningiomas (CSMs). Recent advances in skull base microsurgery and microanatomy have renewed the understanding of PMs and have contributed to the development of detailed definitions and classifications. Graillon and Mariniello (3, 4) enlarged the definition of PMs and classified them into more subtypes. Individualized surgical strategies were developed and applied to the treatment of PMs on the basis of classifications, to facilitate gross total resection and neurofunction preservation.

In the last 20 years, the widespread use the operating microscope for skull base neurosurgery has allowed neurosurgeons to remove tumors aggressively. However, owing to their localization, PMs often compress, encase, or even invade cranial nerves, skull base bones, cerebral dura mater, cerebral arteries, and their perforating branches, thus resulting in unsatisfactory total tumor resection. The past few years have witnessed the development of stereotactic radiotherapy and molecular targeted therapy for meningiomas, which tempered the enthusiasm for aggressive total resection and influenced surgical strategies to be more conservative. To protect neurovascular structures during operation, the purpose of PM microsurgery is maximal surgical resection followed by adjuvant therapy. The dissection of the cerebral arteries and their perforating branches that are encased or invaded by PMs is the key surgical technique for achieving maximal tumor resection.

The identification of PMs subtypes before surgery is of great significance in individualized cerebral artery protection strategies. With the advances in preoperative neuroimaging, we were able to extend the classification of Ugrumov and classified PMs into ACMs, CMSs, and MSWMs before surgery. These subtypes originate from adjacent regions are difficult to distinguish from each other when they extensively invade surrounding structures. In the current study, we present a method for distinguishing three PMs based on preoperative imaging and through which we can apply the individualized surgical approach to patient. Concurrently, a bidirectional dissection technique was performed to achieving maximum tumor resection while preserving cerebral arteries and their perforating branches.

## Methods

### Patient Population

We retrospectively analyzed neuroimaging, intraoperative video, and follow-up data from a consecutive series of 163 patients with PMs. The patients included in this study underwent microsurgery between January 2012 and March 2020 in the Department of Neurosurgery, XiangYa Hospital Central South University. The surgery was performed by the senior author Qing Liu. All patients underwent MRI, computed tomography angiography (CTA), KPS, muscle strength grading, and neurological examination before and after surgery. PMs were confirmed again by pathological examination, and the dural origin was observed during the operation. Meningiomas originating from the petroclival region, middle skull base, sellar region, and suprasellar region with secondary involvement of the parasellar region were excluded.

### Imaging and Tumor Classification

**Figure 1:**
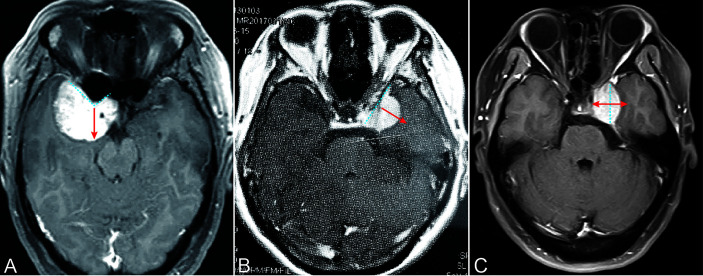
Extension directions of three typical PCMs. **(A)** ACMs extended to the direction in which the anterior clinoid projected. **(B)** MSWMs grow perpendicular to the sphenoid ridge. **(C)** CSMs extended perpendicular to the lateral wall of the cavernous sinus. Cyan dash line: the base of the tumor. Red arrow: tumor extension directions.

All patients underwent routine MRI before surgery, which include T1- and T2-weighted MRI with and without Gd. By using preoperative imaging, we can easily classify PMs into three subtypes (**Figure 1**) according to their growth directions and involved structures. On the basis of imaging classification, an individualized surgical approach and bidirectional dissection technique would be performed. In addition, the degree of resection can be estimated before surgery. For example, the total resection of CSMs with extensive intracavernous extension entails a high risk of cranial nerve injury. Instead of aggressive total resection, we removed the tumor with the goal of maximal resection while preserving the neurofunction. The residual tumor would be required for stereotactic radiotherapy three months after surgery.

We classified these cases as ACMs, CSMs, and MSWMs on the basis of radiological features of preoperative imaging. Consistent with our experience and other reports (5), ACMs originate from the dura covered in the inferior, superior and lateral aspects of the anterior clinoid (6). Coronal MRI images showed that the epicenter of ACMs was located on and around the anterior clinoid and formed a “v” shape. ACMs often extended to the direction which anterior clinoid projected to (**Figure 1A**). By contrast, MSWMs tended to grow perpendicular to the sphenoid ridge and compress the temporal lobe (**Figure 1B**). The other important features of ACMs that we can conclude from CTA or high-resolution computed tomography (HRCT) were the anterior clinoid hyperostosis and optic canal involvement. By contrast, MSWMs and CSMs rarely extended to those structures. CSMs represent a kind of meningiomas that originate from arachnoid granulations localized to the intermembrane space of the lateral wall of the cavernous sinus (7), and CSMs that originate from the outside of the cavernous sinus and those that secondarily invaded into it were excluded. We observed that the CSMs tended to extend within the cavernous sinus in the early stage because of the dense lateral wall (as reflected in the coronal images) and that the CSMs extended perpendicular to the lateral wall of the cavernous sinus (**Figure 1C**). CN III dysfunction in the early stages, along with the radiological features of CSMs, allow surgeon to diagnosed CSMs accurately. Furthermore, large ACMs and MSWMs may secondarily extend to the cavernous sinus *via* different routes. We observed 20 ACMs invading the cavernous sinus through the oculomotor triangle or infiltrating the roof or lateral wall directly. In contrast to ACMs and CSMs, MSWMs seldom invaded the cavernous sinus instead of compressing its lateral wall.

CTA was performed to estimate the relationship between the cerebral arteries and tumors. On the basis of preoperative imaging and intraoperative observation, we divided the cases into three groups: Group A involves tumor-compressed arteries or perforating branches but with the intervening arachnoid plane was intact. Group B involves tumor-displaced or tumor-encased arteries or their perforating branches but with the intervening arachnoid plane was intact; Group C involves tumor-invaded, tumor-encased, or tumor-displaced arteries or their perforating branches, as well as tumors that invaded the adventitia and caused the absence of the intervening arachnoid plane. HRCT was used to evaluate hyperostosis or bone erosion.

### Surgical Approach

The classic pterional approach (frontotemporal approach) has been widely applied to PM surgery for several decades. Our individualized surgical strategies were based on this approach and its modified form. A total of 105 of 163 craniotomies (64.4%) were performed *via* the pterional approach, and the remaining 58 craniotomies were performed using the pretemporal transcavernous approach. A brief overview of our tailored surgical approach based on preoperative imaging is provided below.

When surgery was performed using the pterional subdural approach, the incision was initiated above the palpated zygoma, extending superiorly and then curving anteriorly from the superior temporal line to the limit of the contralateral hairline. Subsequently, a standard frontotemporal craniotomy was performed. The drilling of outer and middle portions of the sphenoid wing was followed tby craniotomy. After the meningoorbital band was transected, drilling was continued to remove the anterior clinoid or optic canal if the tumor invaded those structures. A pterional intradural approach was required to open the dura mater after the extradural steps were completed. The dura was opened in a semicircular incision centered on the Sylvian fissure and extended inferior to the floor of the anterior and middle skull base. The arachnoid membrane over the sphenoid wing was opened to allow the drainage of cerebrospinal fluid (CSF) and the elevation of the temporal lobe. The dissection plane was established using the operation microscope, and the tumor was removed by bipolar coagulation and suction in small parts.

The pretemporal transcavernous approach required the same incision and craniotomy as the pterional subdural approach, but it did not necessitate entry into the subdural space in most cases. The surgery began by dissecting the meningo-orbital band with a microdissector, which can be used to peel off the outer layer of the lateral wall of the cavernous sinus. An incision parallel to the oculomotor or trochlear nerve was made in the inner layer of the lateral wall to provide entry into the cavernous sinus. The tumor within the cavernous sinus was removed by suction. A residual tumor encasing the ICA or its branches was also removed using bidirectional dissection technology. When a tumor that extended to the medial part of the cavernous sinus or petroclival region was encountered, we combined the pterional intradural approach and dissection of the Sylvian fissure. The dura mater of the superior wall of the cavernous sinus was opened, and we were able to access the residual tumor localized to the inner space of the ICA *via* the Dolenc triangle. By following the superior wall and proceeding posteriorly along the tentorial incisura, the tumor extending to the upper part of the petroclival region was removed.

### Bidirectional Dissection

**Figure 2:**
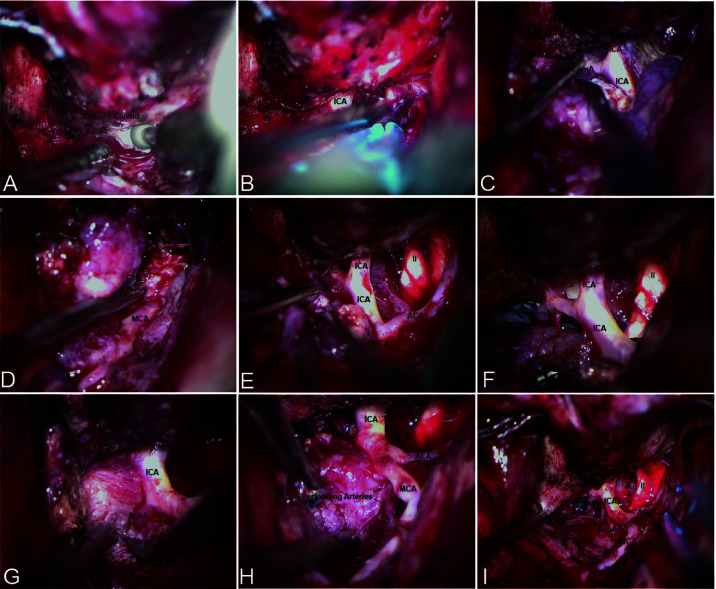
Bidirectional dissection technique applied in ACM. **(A)** Extradural anterior clinoidectomy. **(B)** The proximal ICA can be localized in the medial of the anterior clinoid. **(C)** Forward dissection was started from the identification of the proximal ICA and along the course of the ICA. **(D)** Reverse dissection was initiated after split the Sylvian fissure. **(E–H)** Forward dissection would be applied again after debulking and resection most of the tumor. **(I)** Total resection and artery protection can be achieved by bidirectional dissection.

In patients with ACMs and large MSWMs, bidirectional dissection began with extradural anterior clinoidectomy (**Figure 2A**), and the ICA and optic nerve (ON) that localizes to the medial of the anterior clinoid were determined (**Figure 2B**). Forward dissection was performed to expose the proximal ICA. Dissection proceeded from the distal dura ring of the ICA to its bifurcations (**Figure 2C**). Reverse dissection was initiated from the debulking tumor extending to the surface of the Sylvian fissure, and the dissection plane between the tumor and neurovascular structures was identified. The distal MCA branches can be located after splitting the Sylvian fissure. Tumor dissection was started from the MCA toward the ICA by using a microdissector, a microscissor, and a suction in small pieces (**Figure 2D**). When the tumor invaded the adventitia or was too hard to suction, sharp dissection using a no. 11 blade scalpel was used to remove the tumor covering the arteries. Reverse dissection may be hindered by meningioma calcification or perforating branches, and forward dissection can be applied again to remove the residual tumor (**Figure 2E**). Following the course of the ICA trunk, the MCA, ACA, and anterior choroidal artery (ACHA) can be easily dissected from the residual tumor easily (**Figures 2F, G**). Special care must be taken to identify the perforating branches of the ICA and MCA embedded in the tumor (**Figures 2H, I**).

**Figure 3:**
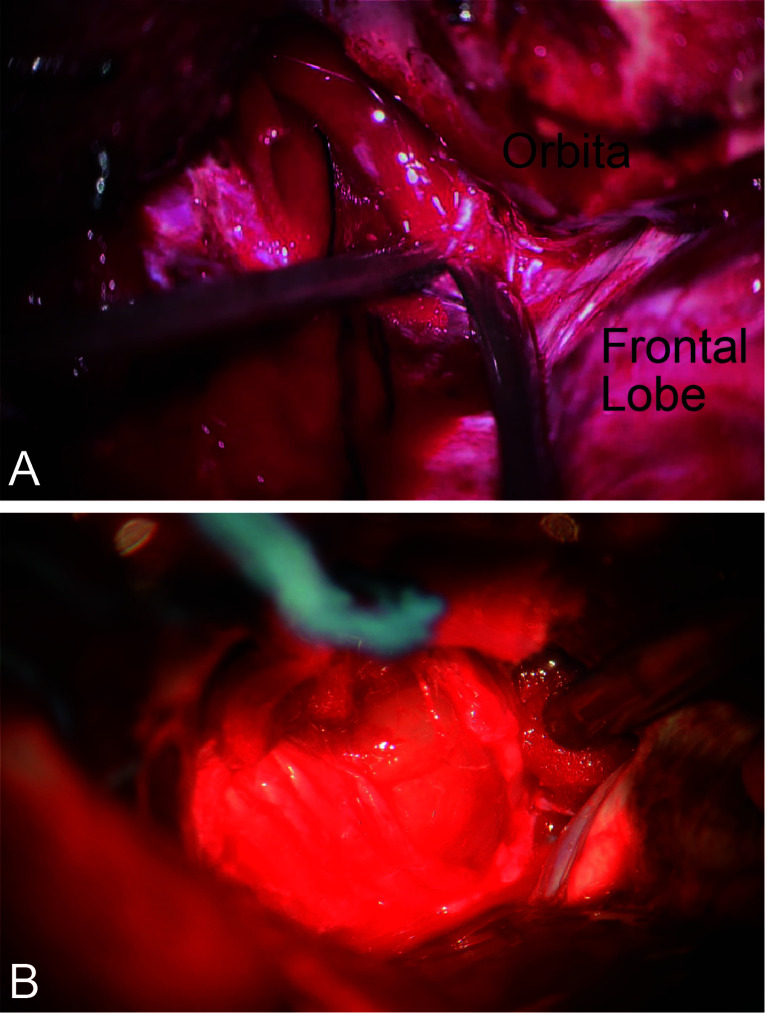
Bidirectional dissection technique applied in CSM. **(A)** Dissection by peeling off the outer layer of the lateral wall and incising the inner layer of the cavernous sinus. **(B)** The cranial nerve coursing through the outer layer of the lateral wall.

For patients with CSMs, forward dissection started with the identification of the meningo-orbital band, followed by the peeling of the outer layer of the lateral wall and the incising of the inner layer of the cavernous sinus (**Figure 3A**). Opening the space between the trochlear nerve and the ophthalmic division can expose the posterior bend and horizontal segment of the intracavernous ICA. The abducent nerve coursing through the lateral surface of the ICA should be protected (**Figure 3B**). The posterior bend is a landmark of reverse dissection from which the meningohypophyseal trunk is obtained. The trunk is the most important perforating branch of the intracavernous ICA and should be preserved using the bidirectional dissection technique. The dissection proceeded from the posterior bend forward to the horizontal segment of the ICA *via* suction and microdissection. However, limited by the ICA and trochlear nerve, forward dissection cannot be applied to the resection of the tumor in the inner part of the ICA. Reverse dissection was initiated by opening the Dolenc triangle of the superior wall of the cavernous sinus on the basis of the intradural approach, by localizing the proximal dura ring of the ICA, and by dissecting the tumor from the anterior ascending segment to the anterior bend of the ICA.

## Results

### Patient Population

From January 2012 to March 2020, 163 PMs (63 ACMs, 58 CSMs, and 42 MSWMs) were surgically removed by the senior authors. Among the patients, 113 were female (69%), and 50 were male (31%). The median age was 52.5 years. The most common presenting symptoms were headache (53.2%), visual impairment (42.8%), visual field defect (26.6%), diplopia (17.2%), hemiparesis (6.8%), seizures (5.0%), oculomotor paralysis (14.3%), abducent paralysis (18.1%), and trochlear nerve palsy (12.5%). The mean follow-up time in our study was 38.8 months.

### Imaging and Intraoperative Findings

PM classification was based on preoperative radiological characteristics and intraoperative inspection. A total of 63 tumors with a meningioma epicenter of the anterior clinoid growing toward the direction where the anterior clinoid extends were classified into ACMs (**Figure 1A**). A total of 42 tumors with meningioma originating from the dura of the inner medial sphenoid wing and growing perpendicular to the long axis of the sphenoid wing to compress the medial temporal lobe were grouped into MSWMs (**Figure 1B**). The remaining 58 tumors, which originated within the cavernous sinus or the lateral wall and grew perpendicular to the long axis of the cavernous sinus, were grouped into CSMs (**Figure 1C**).

The prominent imaging and intraoperative findings showed the involvement of the tumor with the ICA, MCA, ACA, ACHA, and posterior communicating artery (PcomA). According to our preoperative or intraoperative observation, ICA and its branches were encased or invaded in most patients with PMs, whereas ACHA and PcomA were only involved in large PMs (**Table 1**).

**Table 1:** The relationship between the tumor and cerebral arteries in different PMs.

	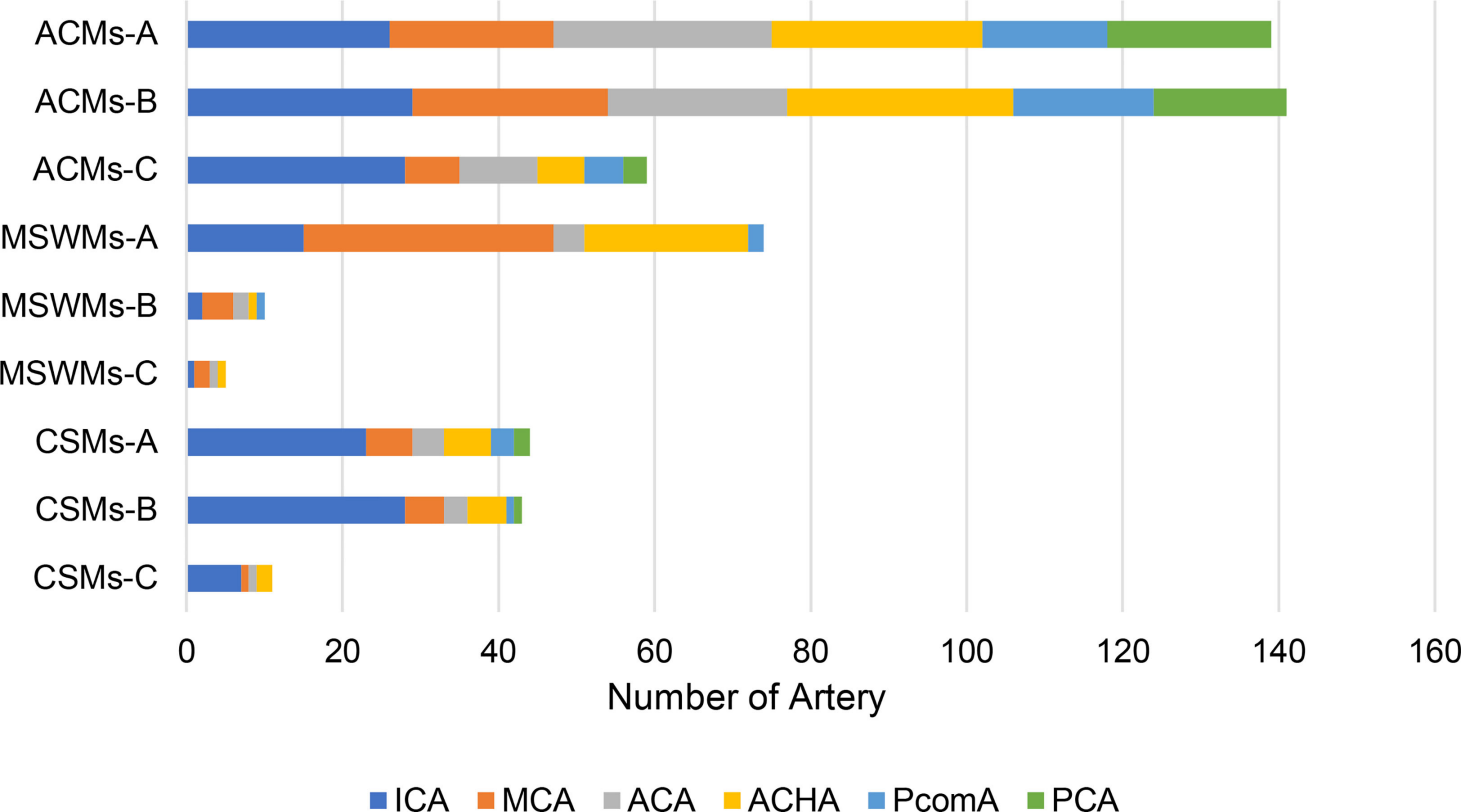	

ICA was involved in most of ACMs and CSMs while MCA tend to be compressed or invaded by most of the MSWMs.

The important structures of the parasellar region have also been found to be involved in tumors. ON impressment or encasement and optic canal involvement were found in most ACMs, whereas CSMs and MSWMs seldom invaded them. Cavernous sinus involvement was found in almost all CSMs and in some ACMs that invaded into the cavernous sinus *via* the oculomotor triangle or invaded the wall of the cavernous sinus directly. MSWMs can hardly invade the cavernous sinus, and they tend to compress the lateral wall of this area. The superior orbital fissure was mainly invaded by MSMWs.

### Surgical Results

Tailored approaches were applied according to the preoperative imaging classification. The pterional intradural approach was the most frequently performed approach for 63 ACMs and 42 MSWMs. The pretemporal transcavernous approach was performed in 58 patients with CSMs.

Total resection (Simpson grade I or II) was achieved in 50 patients with ACMs (79.3%), 38 patients with MSWMs (90.5%), and 26 patients with CSMs (44.8%). Subtotal resection (Simpson grade III) and partial resection (Simpson grade IV) were achieved in 13 patients with ACMs (20.7%), 4 patients with MSWMs (9.5%), and 32 patients with CSMs (55.2%) (**Table 2**). The tumor invaded into the cavernous sinus or the adventitia of the artery, and tumor calcification and tumor adherence to the epineurium were the three main reasons for subtotal or partial resection.

**Table 2:** Bar graph showing the extent of resection in three types PCMs.

	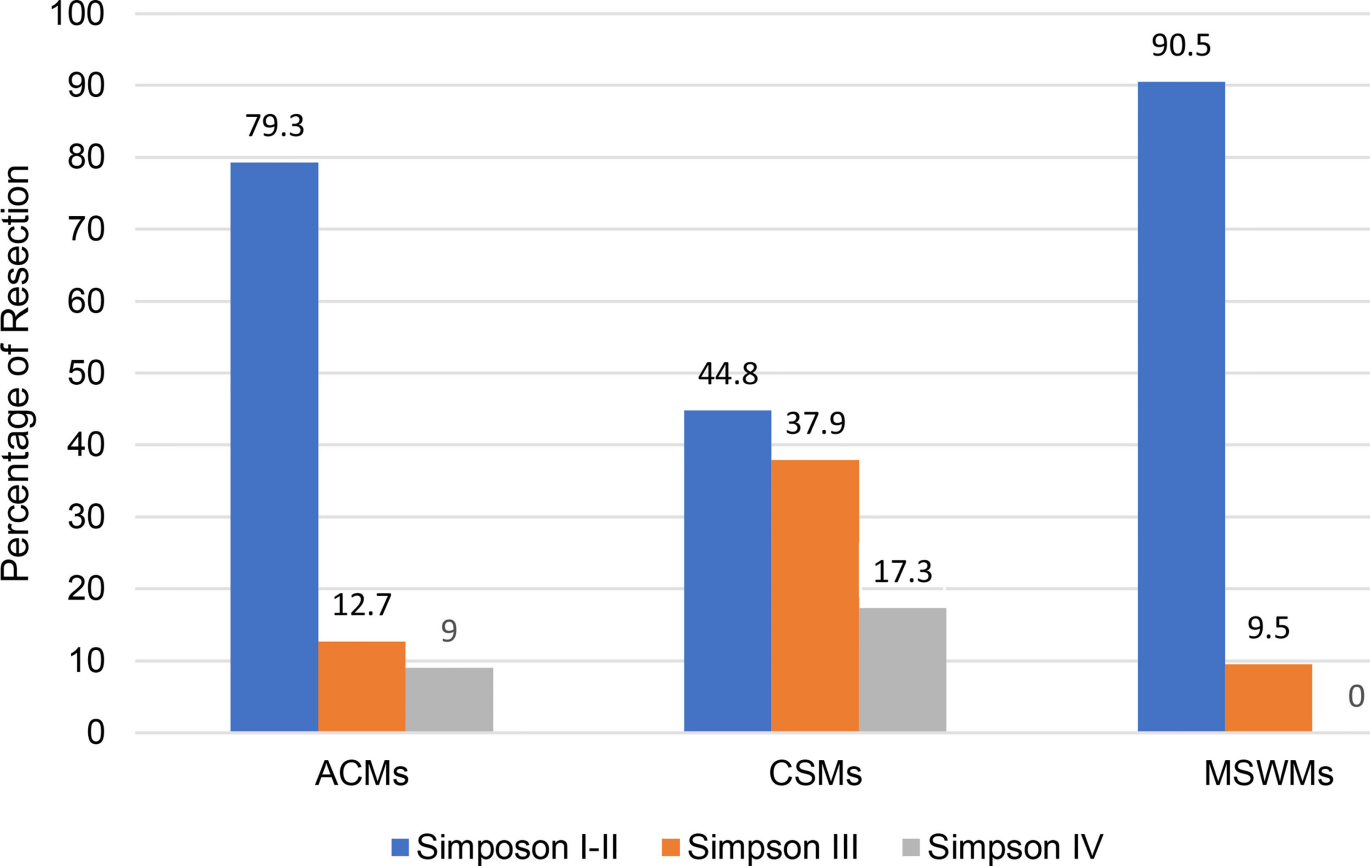	


Histopathology confirmed that 5 patients (3.1%) had WHO grade II meningiomas, whereas 158 patients (96.9%) had WHO grade I meningiomas. Even though total resection was achieved, the 5 patients with WHO grade II meningioma required radiotherapy after three months.

Postoperative computed tomography scan were performed on all patients after recovery from anesthesia to evaluate the extent of tumor resection and postoperative complications. Postoperative cerebral hemorrhage and severe encephaledema were found in 10(7.7%) and 13(8.0%) patients, respectively, owing to our vascular protection strategy. Patients were required to undergo contrast enhanced MRI to confirm the extent of resection again 72h after the surgery. We suggested that patients with residual tumors initiated stereotactic radiotherapy after three months.

### Neurofunctional Outcome

Postoperative neurofunction outcomes were defined as improved, unchanged, or deteriorated (**Table 3**). Preoperative and postoperative neurofunction was evaluated by ophthalmologic examination or the MRC scale for muscle strength grading at the first follow-up after 3 months.

**Table 3:** The number of patients with CN function and myodynamia change at the latest follow-up.

		CN II	CN III	CN IV	CN VI	Myodynamia
ACMs	improved	26	23	16	29	20
	unchanged	35	36	45	33	43
	deteriorated	2	4	2	1	
CSMs	improved	20	25	27	29	22
	unchanged	35	31	28	27	36
	deteriorated	3	2	3	2	
MSWMs	improved	12	19	16	15	18
	unchanged	29	23	36	27	34
	deteriorated	1				

The visual acuity improvement rates in patients with three types of meningiomas were 41.3%, 46.5%, and 47.6% for ACMs, CSMs, and MSWMs, respectively. However, the vision acuity of most patients remained unchanged at follow-up several months postoperatively.

CN III deterioration was the most common type of CN deterioration in all patients. Four patients with ACMs and two patients with CSMs suffered from transient CN III deterioration, and most of them recovered to unchanged status after a few months. CN IV and CN VI deteriorations were mainly found in five postoperative patients with CSMs (**Table 3**).

### Tumor Recurrence

Overall, 155 patients (95%) underwent long-term follow-up. The actual follow-up time ranges from 11.9–65.7 months. Follow-up was performed with contrast-enhanced MRI. Tumor recurrence or progression was observed in eight patients with CSMs, two patients with ACMs, and one patient with MSWMs. A total of 32 patients with residual tumor within the cavernous sinus or residual tumor that invaded the artery remained stable. The mean KPS performed in patients at follow-up was 89.6 compared with 81.1 preoperatively.

## Discussion

In this study, we improved the classification of Ugrumov (2) and grouped PMs into three subtypes on the basis of preoperative imaging. The classification allowed us to perform individualized surgical approaches and artery protection strategies to achieve maximum tumor resection and minimized mobility. According to Giordano (8), intraoperative MRI allows surgeons to better evaluate the resectability of PCMs. However, in our experiences, we were able to evaluate the resectability of different PCMs before surgery instead of refering to the time-consuming intraoperative MRI.

### Individualized Surgical Strategies

Surgical approaches for removing PMs have been published in many reports. The traditional approach for PM surgery was the pterional approach which was first described by Dandy (9) in 1942. Dolenc (10) applied the pterional approach to the surgical treatment of cavernous hemangioma of the cavernous sinus. In the last 20 years, pterional approach has been widely applied to the surgical treatment of PMs (5, 11–15), and the pterional approach and extended pterional approach have been centered around the Sylvian fissure with exposure of the temporal and frontal lobes. After removing the bone flap, there are two main routes to the parasellar region. The intradural approach is the most widely used method. After dural opening and CSF drainage, the temporal lobe can be easily elevated to expose the tumor. Another approach is the extradural approach, in which a dural incision is not required. Instead, by removing the sphenoid wing and dissecting the meningo-orbital band, surgeons can easily enter the intermembrane space of the lateral wall of the cavernous sinus to remove the tumor. In this series, we present our experience by combining intradural and extradural approaches to the parasellar region *via* pterional or extended pterional craniotomy.

For ACMs and MSWMs, we drilled the bone constituting the sphenoid wing and transected the meningoorbital band. Thereafter, we elevated the dura from the extradural area and conjugated the arterial supply from the ophthalmic artery, the anterior branch of the middle meningeal artery and the meningo-orbital artery (13, 16). These techniques have the following advantages: (1) Bone removal and elevation of the dura allow us to identify the ON and ICA from the extradural area. (2) The interruption of the arterial supply from the extradural area alleviates bleeding in the intradural procedures. (3) Bone removal enlarges the operation room and reduces temporal lobe retraction. To alleviate the tension of the brain, we chose to open the dura mater for CSF drainage before tumor resection. A large tumor would be pushed to the surgeon after splitting the Sylvian fissure with the help of the high CSF pressure of the basal arachnoid cisterns. Tumor debulking was initiated from the outside to the inside. By using the bidirectional dissection technique, we can remove the residual tumor that encases the ICA or MCA in small pieces. Anterior clinoidectomy was performed in patients with anterior clinoid hyperostosis or erosion to minimize intraoperative complications. We prefer extradural clinoidectomy because the dura mater acts as a protective screen during dissection or drilling. In addition to this advantage, extradural clinoidectomy allows a section of the falciform ligament to decompress the ON and protect it from the compression of the sharp falciform ligament. Owing to these procedures, visual function deterioration was observed in 2.9% of patients with ACMs and MSMWs.

For CSMs, the bone of the sphenoid wing was drilled to the base of the middle cranial fossa to enlarge the dissection space. Dissection into the cavernous sinus was initiated from the meningo-orbital band, which is located at the apex of the superior orbital fissure (**Figure 3A**). By performing a sharp dissection, a plane between the temporal tip and cavernous sinus lateral wall was established. The outer layer of the lateral wall was peeled away from the anterior aspect of the cavernous sinus (**Figure 3B**). The tumor invading the intermembrane space of the lateral wall can be removed by suction and sharp dissection. An incision parasellar to the CN IV was made in the enlarged space caused by tumor compression superior or inferior to the CN IV to provide entry the cavernous sinus. These maneuvers took advantage of the natural space between the CNs caused by tumor compression and enabled the surgeon to enter the cavernous sinus *via* the shortest routes. Sharp dissection along the cleavage planes within the cavernous sinus minimized injury to the CNs and arteries caused by traction or suction. When the tumor invaded the inner part of the cavernous sinus, we performed the intradural approach and entered the cavernous sinus *via* an incision in its roof. This procedure enabled the total resection of CSMs. When the tumor invaded the adventitia or epineurium of the cavernous sinus, we left the residual tumor in this area and then performed postoperative stereotactic radiotherapy. The recurrence and progression rate of CSMs was 13.7% compared with 7.5%-20% reported in other studies (11–13). A few patients exhibited temporary postoperative cranial dysfunction, and most of them recovered to preoperative levels after several months.

### Bidirectional Dissection

**Figure 4:**
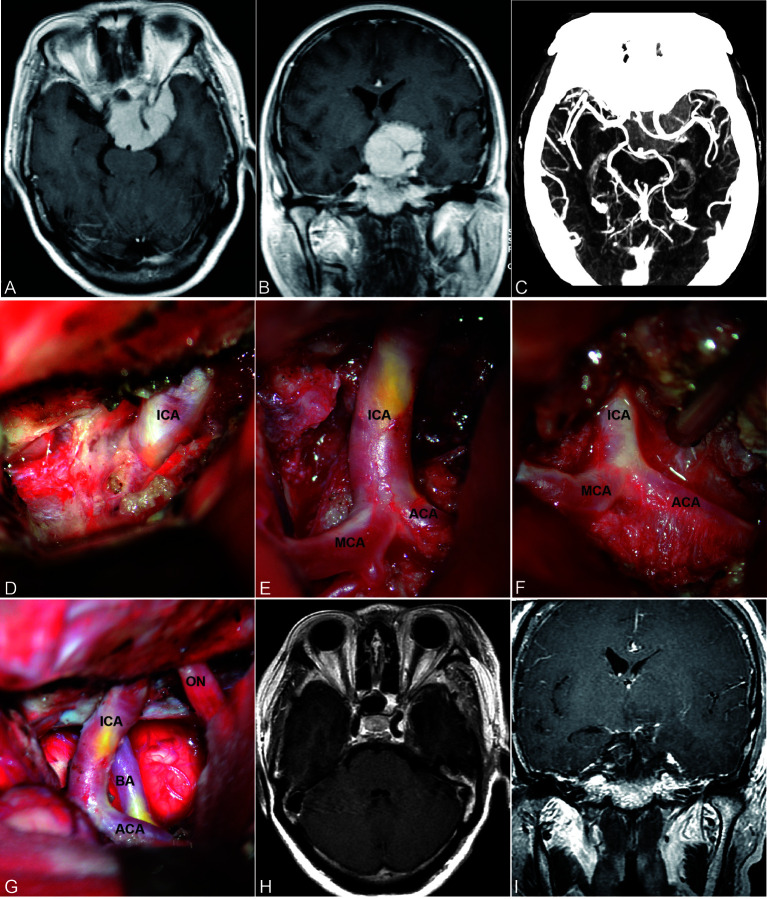
Contrast-enhanced MRI and CTA images of ACM **(A–C)** preoperative; **H, I** postoperative) and steps of bidirectional dissection technique **(D–G)**. Intradural localization of the ICA **(D)** and dissection along the course of ICA and its branches **(E, F)** to achieve total resection while preserved the ICA and its branches **(G)**.

The encasement of the ICA and its branches has been reported in 20%-55% (13, 14). Vessel invasion is dealt with aggressively, and unintended vessel perforation occurs in 20.8% of patients (6). Sacrificing the middle cerebral artery branches invaded by ACMs resulted in 100% mortality (17). PMs that encase or invade the major cerebral arteries and their perforating branches remain challenging for total surgical resection. However, residual tumor along the arteries is a risk factor for tumor progression. Maximal tumor resection while preserving the arteries is the desired surgical goal. To achieve this goal, we performed a bidirectional dissection technique based on a tailored surgical approach (**Figure 4**).

On the basis of the individualized preoperative classification and approach, we performed this technique in different directions, and one patient suffered from artery perforation. The postoperative hemiparesis rate was 3.1% in patients with PMs. This technique facilitates tumor debulking and differentiation of the feeding artery or perforators. Sharp dissection combined with mild traction is the core technique for bidirectional dissection. Compared with suction and blunt dissection, sharp dissection along the arachnoid plane prevents traction force from injuring CNs and perforating arteries. Once the tumor invaded the adventitia or epineurium, the arachnoid membrane was absent. This led to arteriotomies being indicated on preoperative imaging even though the tumor was small. We chose to leave the tumor tuft along the arteries and their perforating branches.

**Figure 5:**
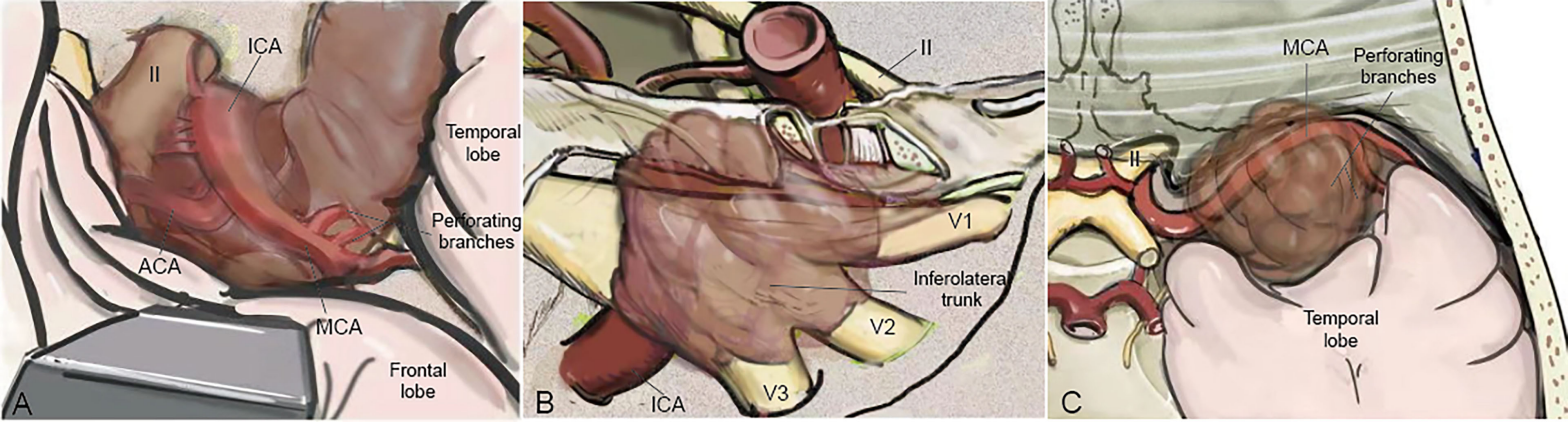
The cranial artery, perforating branches, and the cranial nerve are involved in the PCMs. The ACA, ICA, MCA, and their perforating branches were involved in ACMs **(A)** while MCA and its perforating branches were the most involved in MSWMs **(C)**. The inferolateral trunk and the meningohypophyseal trunk of the ICA were encased by CSMs **(B)**.

The bidirectional dissection technique can be applied to the dissection of the ICA, MCA, ACA, ACHA, and PcomA (**Figure 3**). However, it can also be applied to the dissection of perforating branches. Special attention was given to perforating branches that support CNs (**Figure 5**). The arterial supply of the chiasm and ON is mainly from the superior hypophyseal arteries, which are often damaged by intraoperative operations, thus resulting in subsequent ON ischemia and visual deficits (18). In the current study, we dissected this branch by finding the entrance point from the posteromedial aspect of the ICA and dissecting it from proximal to distal (**Figure 5A**). The meningohypophyseal trunk and inferolateral trunk are the most important branches of the cavernous ICA and can be located in the posterior and horizontal ICA segments, respectively (**Figure 5B**). The sacrifice of these arteries may cause the dysfunction of CN III, IV, and VI because of the interruption of their blood supply (19). By following their course and using the bidirectional dissection technique, these CNs can be protected while achieving maximal tumor resection. The lenticulostriate arteries are the most important perforating branches of the MCA and may cause hemiparesis, coma or death when damaged (20). The majority of these perforating branches coursed medially and parallel to the M1 supply of the basal ganglia and portions of the internal capsule. Special attention is needed to protect this branch during dissection along the MCA trunk. The dissection of the lenticulostriate arteries was initiated from the MCA trunk followed by tumor decompression to identify the distal end of the lenticulostriate arteries encased in tumors (**Figure 5C**). By using these bidirectional dissection procedures, maximal tumor resection and neurofunctional protection can be achieved.

### Neurofunctional Outcomes at Long-Term Follow-Up

During the past two decades, our surgical goal was to achieve maximal tumor resection while protecting the neurofunction of patients and improving their postoperative quality of life. The individualized surgical techniques mentioned above were used to minimize mobility and morbidity. In our series, the KPS of patients with PMs at the last follow-up was elevated to 89.6 compared with 81.1 preoperatively.

The ON was often involved in PMs and postoperative visual improvement was present in 10%-66.7% of patients according to the previous reports (6, 14, 17). The visual acuity improvement and unchangeability in our series were up to 90.3%. We attributed the good outcome not only to the bidirectional dissection technique but also to the extradural clinoidectomy, the section of the falciform, and the preservation of the perforating branches that supply the ON and optic chiasm. In our experience, the compression of the nerve directly or its feeding artery was the reason for the deterioration of visual acuity preoperatively. Even in blind patients, efforts should be made to dissect the ON and its feeding arteries from the tumor instead of sacrificing it to achieve maximal tumor resection. Three patients with blind-eye recovery had a sense of light after several months. Another important procedure that may contribute to the improvement of patients is the dissection of the superior hypophyseal arteries, which is the main blood supply of the ON.

CN III, IV, and VI are often encased or invaded by CSMs and large ACMs. Injury to these CNs or injury to their blood supply were the two main reasons for postoperative CN dysfunction. According to previous reports, postoperative CN III, IV, and VI dysfunction from 12.9% to 29% (11–13, 21). The deterioration of nerve function was observed in 9.2% of our patients, and 66.7% recovered to unchanged after follow-up. We attributed these results to the following individualized surgical techniques: (1) A pretemporal transcavernous approach allows the surgeon to identify the plane between the tumor and neurovascular structures within the cavernous sinus. (2) Sharp dissection minimizes the traction to CNs. (3) A bidirectional dissection technique was used to preserve the blood supply of these CNs. The total resection rate of CSMs was 44.8%, which was lower than that of ACMs and MSWMs. The reason was to prevent intraoperative injury to the ICA and CNs when the tumor invaded the adventitia or epineurium. If we remove the residual tumor in the above cases, injury to the ICA or CNs was unavoidable. Owing to the slow growth of the residual tumor (22), we left the residual tumor and performed stereotactic radiotherapy three months after the surgery. The recurrence rate of CSMs was 3.4% during the follow-up.

### Limitations

Most of the results of this study were based on retrospective data. This retrospective aspect may introduce selection bias and misclassification. Prospective studies and multi-organizational research with larger sample sizes are still needed before the surgical techniques and results of this study can be adopted.

## Conclusion

PMs are one of the most challenging skull base meningiomas. We modified the classification of Ugrumov (2) and classified PMs into three subtypes on the basis of preoperative imaging, according to which we can perform individualized surgical strategies for patients, including tailored surgical approaches and bidirectional dissection techniques. This technique contributes to the total resection of meningiomas while preserving the cerebral arteries and CNs.

## Data Availability Statement

The original contributions presented in the study are included in the article/**Supplementary Material**. Further inquiries can be directed to the corresponding author.

## Ethics Statement 

The studies involving human participants were reviewed and approved by the Ethics Committee of Xiangya Hospital. The patients/participants provided their written informed consent to participate in this study.

## Author Contributions

YL analyzed and interpreted the patient data regarding the PCMs and drafted the article. XZ collected the patient data. CQ and JS provided critical revise. KX and XW draft the figures. QL and other authors were performed the surgeries. All authors contributed to the article and approved the submitted version.

## Funding

This work was supported by grants from the National Key Technology Research and Development Program of the Ministry of Science and Technology of China (grant number 2014BAI04B01).

## Conflict of Interest

The authors declare that the research was conducted in the absence of any commercial or financial relationships that could be construed as a potential conflict of interest.

## Publisher’s Note

All claims expressed in this article are solely those of the authors and do not necessarily represent those of their affiliated organizations, or those of the publisher, the editors and the reviewers. Any product that may be evaluated in this article, or claim that may be made by its manufacturer, is not guaranteed or endorsed by the publisher.

## Supplementary Material

Supplementary Video 1 Forward dissection was performed to expose the proximal ICA. Dissection proceeded from the distal dura ring of the ICA to its bifurcations Reverse dissection was initiated from the debulking tumor extending to the surface of the Sylvian fissure. The distal MCA branches can be located after splitting the Sylvian fissure. When the tumor invaded the adventitia or was too hard to suction, sharp dissection using a no. 11 blade scalpel was used to remove the tumor covering the arteries. Following the course of the ICA trunk, the MCA, ACA, and anterior choroidal artery (ACHA) can be easily dissected from the residual tumor easily.

## References

[1] Stirling W. Physiology in the French Metropolis. Br Med J (1896) 1(1841):923–4. doi: 10.1136/bmj.1.1841.923 PMC2406949 20756150

[2] Ugrumov VM Ignatyeva GE Olushin VE Tigliev GS Polenov AL. Parasellar Meningiomas: Diagnosis and Possibility of Surgical Treatment According to the Place of Original Growth. Acta Neurochir Suppl (Wien) (1979) 28(2):373–4. 290210

[3] Graillon T Regis J Barlier A Brue T Dufour H Buchfelder M. Parasellar Meningiomas. Neuroendocrinology (2020) 110(9-10):780–96. doi: 10.1159/000509090 32492684

[4] Mariniello G de Divitiis O Bonavolonta G Maiuri F. Surgical Unroofing of the Optic Canal and Visual Outcome in Basal Meningiomas. Acta Neurochir (Wien) (2013) 155(1):77–84. doi: 10.1007/s00701-012-1485-z 22945895

[5] Pamir MN Belirgen M Ozduman K Kilic T Ozek M. Anterior Clinoidal Meningiomas: Analysis of 43 Consecutive Surgically Treated Cases. Acta Neurochir (Wien) (2008) 150(7):625–35; discussion 35–6. doi: 10.1007/s00701-008-1594-x 18509587

[6] Al-Mefty O. Clinoidal Meningiomas. J Neurosurg (1990) 73(6):840–9. doi: 10.3171/jns.1990.73.6.0840 2230967

[7] Kehrli P Maillot C Wolff Quenot MJ. Sheaths of Cranial Nerves in the Lateral Wall of the Cavernous Sinus. An Embryological and Anatomical Study. Neurochirurgie (1995) 41(6):403–12. doi: 10.1179/tns.1993.001 8815415

[8] Giordano M Gallieni M Metwali H Fahlbusch R Samii M Samii A. Can Intraoperative Magnetic Resonance Imaging Be Helpful in the Surgical Resection of Parasellar Meningiomas? A Case Series. World Neurosurg (2019) 132:e577–84. doi: 10.1016/j.wneu.2019.08.070 31442639

[9] Dandy WE. Aneurysm of the Anterior Cerebral Artery. J Am Med Assoc (1942) 119(16):1253–4. doi: 10.1001/jama.1942.72830330001005

[10] Dolenc V. Direct Microsurgical Repair of Intracavernous Vascular Lesions. J Neurosurg (1983) 58(6):824–31. doi: 10.3171/jns.1983.58.6.0824 6854374

[11] Nanda A Thakur JD Sonig A Missios S. Microsurgical Resectability, Outcomes, and Tumor Control in Meningiomas Occupying the Cavernous Sinus. J Neurosurg (2016) 125(2):378–92. doi: 10.3171/2015.3.JNS142494 26745483

[12] Pichierri A Santoro A Raco A Paolini S Cantore G Delfini R. Cavernous Sinus Meningiomas: Retrospective Analysis and Proposal of a Treatment Algorithm. Neurosurgery (2009) 64(6):1090–9. doi: 10.1227/01.NEU.0000346023.52541.0A 19487888

[13] Sindou M Wydh E Jouanneau E Nebbal M Lieutaud T. Long-Term Follow-Up of Meningiomas of the Cavernous Sinus After Surgical Treatment Alone. J Neurosurg (2007) 107(5):937–44. doi: 10.3171/JNS-07/11/0937 17977264

[14] Attia M Umansky F Paldor I Dotan S Shoshan Y Spektor S. Giant Anterior Clinoidal Meningiomas: Surgical Technique and Outcomes. J Neurosurg (2012) 117(4):654–65. doi: 10.3171/2012.7.JNS111675 22900847

[15] Lynch JC Pereira CE Goncalves M Zanon N. Extended Pterional Approach for Medial Sphenoid Wing Meningioma: A Series of 47 Patients. J Neurol Surg B Skull Base (2020) 81(2):107–13. doi: 10.1055/s-0039-1677728 PMC7082174 32206527

[16] Yamaki T Tanabe S Sohma T Uede T Shinya T Hashi K. Feeding Arteries of Parasellar Meningiomas–Angiographic Study of Medial Sphenoid Ridge and Tuberculum Sellae Meningiomas. Neurol Med Chir (Tokyo) (1988) 28(6):553–8. doi: 10.2176/nmc.28.553 2460782

[17] Goel A Gupta S Desai K. New Grading System to Predict Resectability of Anterior Clinoid Meningiomas. Neurol Med Chir (Tokyo) (2000) 40(12):610–6; discussion 6-7. doi: 10.2176/nmc.40.610 11153190

[18] van Overbeeke J Sekhar L. Microanatomy of the Blood Supply to the Optic Nerve. Orbit (2003) 22(2):81–8. doi: 10.1076/orbi.22.2.81.14316 12789588

[19] Salaud C Decante C Ploteau S Hamel A. Implication of the Inferolateral Trunk of the Cavernous Internal CAROTID Artery in Cranial Nerve Blood Supply: Anatomical Study and Review of the Literature. Ann Anat (2019) 226:23–8. doi: 10.1016/j.aanat.2019.07.004 31330308

[20] Djulejic V Marinkovic S Milic V Georgievski B Rasic M Aksic M. Common Features of the Cerebral Perforating Arteries and Their Clinical Significance. Acta Neurochir (Wien) (2015) 157(8):1393. doi: 10.1007/s00701-015-2462-0 26066534

[21] DeMonte F Smith HK al-Mefty O. Outcome of Aggressive Removal of Cavernous Sinus Meningiomas. J Neurosurg (1994) 81(2):245–51. doi: 10.3171/jns.1994.81.2.0245 8027808

[22] Hashimoto N Rabo CS Okita Y Kinoshita M Kagawa N Fujimoto Y. Slower Growth of Skull Base Meningiomas Compared With Non-Skull Base Meningiomas Based on Volumetric and Biological Studies. J Neurosurg (2012) 116(3):574–80. doi: 10.3171/2011.11.JNS11999 22175721

